# Validation of a self-reported instrument to assess work-related difficulties in patients with migraine: the HEADWORK questionnaire

**DOI:** 10.1186/s10194-018-0914-7

**Published:** 2018-09-10

**Authors:** Alberto Raggi, Venusia Covelli, Erika Guastafierro, Matilde Leonardi, Chiara Scaratti, Licia Grazzi, Marco Bartolini, Giovanna Viticchi, Sabina Cevoli, Giulia Pierangeli, Gioacchino Tedeschi, Antonio Russo, Piero Barbanti, Cinzia Aurilia, Carlo Lovati, Luca Giani, Fabio Frediani, Paola Di Fiore, Francesco Bono, Laura Rapisarda, Domenico D’Amico

**Affiliations:** 1 Neurology, Public Health and Disability Unit, Fondazione IRCCS Istituto Neurologico Carlo Besta, Milan, Italy; 2 e-Campus University, Novedrate, Italy; 3 Headache and Neuroalgology Unit, Fondazione IRCCS Istituto Neurologico Carlo Besta, Milan, Italy; 4 Clinica di Neurologia, Università Politecnica delle Marche, Ancona, Italy; 5 IRCCS Istituto delle Scienze Neurologiche di Bologna, Bologna, Italy; 6 DIBINEM - Alma Mater Studiorum, Università di Bologna, Bologna, Italy; 7 Headache Center, Department of Medical, Surgical, Neurological, Metabolic and Aging Sciences, University of Campania “Luigi Vanvitelli”, Naples, Italy; 8 Headache and Pain Unit, Department of Neurological, Motor and Sensorial Sciences. IRCCS San Raffaele Pisana, Rome, Italy; 9 San Raffaele University, Rome, Italy; 10 Neurology Unit, Headache Center, Ospedale L, Sacco University of Milan, Milan, Italy; 11 Neurological and Stroke Unit Department, Headache Center, ASST Santi Paolo e Carlo, San Carlo Borromeo Hospital, Milan, Italy; 12 Headache Center, Institute of Neurology, Magna Graecia University of Catanzaro, Catanzaro, Italy

**Keywords:** Work, Employment, Disability evaluation, Episodic migraine, Chronic migraine, Medication overuse headache, Validation study

## Abstract

**Background:**

The degree to which work-related difficulties are recognized in headache research is poor and often carried out with inadequate information such as “reduced ability to work as usual”, which do not capture at all the variety of difficulties and the factors that impact over them. The aim of this paper is to present the validation of the HEADWORK questionnaire, which addresses the amount and severity of difficulties in work-related tasks and the factors that impact over them.

**Methods:**

We developed a set of items based on a previous literature review and patients’ focus groups and tested it on a wide set of patients with episodic and chronic migraine attending eight different Italian headache centers. HEADWORK factor structure was assessed with exploratory and confirmatory factor analysis; internal consistency and construct validity were addressed as well.

**Results:**

The validation sample (*N* = 373) was mostly composed of patients with episodic migraine without aura (64.3%) and of females (81%). Factor analysis retrieved two different scales: “Work-related difficulties”, composed of eleven items which explain 67.1% of the total variance, and “Factors contributing to work difficulties”, composed of six items which explain 52.1% of the total variance. Both HEADWORK subscales have good measurement properties, with higher scores being associated to higher disability, lower quality of life, lower productivity, higher headache frequency and pain intensity.

**Conclusions:**

HEADWORK is a 17-item, two-scale questionnaire addressing the impact of migraine on work-related difficulties in terms of difficulties in general or specific skills, and the factors contributing to these difficulties, defined as negative impact on work tasks. It can be used to address disability weights for the purpose of calculating the burden of migraine, and to assess the balance between therapeutic and side effects of medication on productivity.

**Electronic supplementary material:**

The online version of this article (10.1186/s10194-018-0914-7) contains supplementary material, which is available to authorized users.

## Background

Episodic Migraine (EM) and Chronic Migraine (CM) have a considerable impact on patients’ daily lives in terms of personal suffering, reduced quality of life (QoL) and disability [1–7], with female and CM sufferers reporting higher disability [8]. In particular, CM frequently presents with medication overuse headache (MOH): as shown in some literature findings, an average of 62.6% (ranging from 50.5% to 68%) of patients with CM present MOH [9–12] and whether MOH is a consequence or a cause of CM has not been clarified [13]. Migraine disorders determine a considerable burden on societies, which is usually addressed in terms of reduced work productivity and cost [14–18]. The most recent studies on the cost of headache disorders, and of EM and CM in particular, showed that most of the cost of such conditions is due to indirect cost, i.e. to reduced work productivity [14, 15, 19]. When addressing the issue of indirect cost, two elements have to be acknowledged: the lost workdays (absenteeism) and the workdays in which people with migraine worked with reduced productivity (presenteeism). Presenteeism is the main driver of migraine cost and burden: in fact, for each lost workday, patients with EM and CM work three to four days with reduced productivity [20, 21], and the cost associated to presenteeism is higher than that associated to absenteeism [14, 15, 22]. Therefore, addressing presenteeism in terms of both frequency of days and impaired productivity is of importance to measure the burden of EM and CM.

While absenteeism can be addressed with a simple and direct question, presenteeism may involve difficulties with the interpretation of content. In fact, the degree to which migraine headaches impact over work-related tasks can be highly variable and is underlined by three elements: a) headache severity, b) the kind of activity or the multiplicity of activities, that constitute one’s own job profile, and c) the context in which one’s own job is carried out. The last two elements may allow the identification of the different tasks and activities as well of contextual elements of the job in terms of interpersonal relationships, and of physical elements that might act as triggers of migraine headaches. A literature review was specifically devoted to understanding the degree to which work-related difficulties are recognized and considered in headache research [23]. In brief, this review was grounded on a previous work in which the International Classification of Functioning, Disability and Health (ICF) [24] was used as a term of reference to describe a set of difficulties that are relevant to migraineurs. Fourteen topics, that could be referred to difficulties with work-related activities, were transformed into MESH terms and were used to search for relevant publications in which these difficulties were experienced by patients with EM, CM, chronic daily headache or MOH. A total of 23 publications were selected and the results showed that there was poor recognition of the topic of work-related difficulties, which was limited to a restricted set of activities such as problem solving, speaking, driving and on “remunerative employment”. The latter topic was generally expressed in terms of reduced ability to “perform job activities” or of reduced ability to “work as usual”, and the meaning of these definitions was less than clear in available literature. The presence of contextual elements was a completely neglected issue.

The main reason for the paucity of information on this topic is, in our opinion, the lack of patient-reported outcome measures (PROMs) specifically aimed to capture the presence, the severity and the type of work-related difficulties in patients with EM and CM. We therefore launched an initiative to develop a new questionnaire, the HEADWORK Questionnaire. Given the paucity of literature data, we ran a qualitative study with the aim of exploring which were the most relevant difficulties experienced by patients with their work activities and which were the factors that contributed most to these difficulties, getting indications directly from employed patients with EM and CM. In this qualitative study we ran three focus groups with 14 patients, that were asked to discuss the main issues that constitute difficulties with work-related activities and factors that contributed to these activities [25]. The results of this qualitative study showed that 27 were the most relevant themes reported by patients, and that they referred to: activities (e.g. reading, writing, speaking), personal factors (e.g. attention, stress), correlated symptoms (e.g. pain, being numb), and contextual elements (e.g. office, colleagues, noise, light). The joint results of the literature review, and of this qualitative analysis enabled us to define a set of relevant themes which referred to 13 activities and 12 factors impacting on these difficulties that were used to develop the preliminary version of the HEADWORK Questionnaire. The aim of this paper is to validate this new questionnaire and report its measurement properties.

## Methods

### Participants

Adult patients, i.e. 18 or older, were enrolled for the validation study between June 2016 and October 2017 among those attending eight different Italian headache centers. The main inclusion criterion was the clinical diagnosis of one of the different migraine form according to the International Classification of Headache Disorders, 3-beta version (ICHD-3Beta) [26], namely EM without and with aura (i.e. codes 1.1 or 1.2 of the ICHD-3Beta), and CM, with or without associated MOH (i.e. code 1.3 with or without associated code 8.2 of the ICHD-3Beta). When available, headache diaries were also used for diagnostic purposes. The other inclusion criterion was the fact of being currently employed (or on sick leave) as main occupation and being paid for the activity at the time of enrolment, i.e. no students, retired people or people working on a voluntary basis were included. Both outpatients and inpatients were enrolled. Exclusion criteria, evaluated by the treating neurologist (LG and DD at Fondazione Istituto Neurologico C. Besta IRCCS; MB and GV at Università Politecnica delle Marche; SC and GP at IRCCS Istituto delle Scienze Neurologiche di Bologna; GT and AR at University of Campania “Luigi Vanvitelli”; PB and CA at IRCCS San Raffaele Pisana; CL and LG at Ospedale L. Sacco, University of Milan; FF and PDF at San Carlo Borromeo Hospital; FB and LR at Magna Graecia University of Catanzaro) on the basis of patient evaluation and accurate history taking, were the following: a) presence of cognitive impairments hampering protocol completion (e.g. severe attention deficits); b) anamnesis of cerebrovascular diseases or brain tumors; c) psychiatric disorders of psychotic area; d) other clinical comorbidities in which pain might be of similar or higher impact on daily activities as compared to migraine (e.g. rheumatic diseases, low back pain and other musculoskeletal chronic conditions).

Participation to the study was on a voluntary basis and all patients were asked to provide written consent before inclusion. The study was approved by the Ethical Committees of the coordinating center, Neurological Institute C. Besta (protocol approval number 07/2016, January 13, 2016) and subsequently ratified by all participating centers (Università Politecnica delle Marche, Ancona; IRCCS Istituto delle Scienze Neurologiche di Bologna, Bologna; University of Campania “Luigi Vanvitelli”, Naples; IRCCS San Raffaele Pisana, Rome; Luigi Sacco Hospital-University of Milan, Milan; San Carlo Borromeo Hospital, Milan; Magna Graecia University of Catanzaro, Catanzaro).

### Protocol

The protocol included the collection of socio-demographic data, and the administration of self-reported tools: the preliminary 25-item version of the HEADWORK questionnaire, the Migraine Disability Assessment schedule (MIDAS) [27, 28], the World Health Organization 12-items Disability Assessment Schedule (WHODAS-12) [29], and the Migraine Specific Quality of Life Questionnaire (MSQ) [30]. The MIDAS was chosen as it is the most commonly used outcome measure in headache research and it provides useful information on days with headache and average pain severity; the WHODAS-12 was chosen in reason of the approach to conceptualization of difficulties due to health reason (see also below) as limitations due to a health condition. Finally, since we were interested in ascertaining that HEADWORK’s content was closer to a disability than to a QoL measure, we decided to rely on the MSQ as it migraine-specific and it is valid for use both EM and CM [21–23].

In the socio-demographic section, besides common information on gender, age, marital status and education, a set of employment-related items were included. Specifically, patients were asked on the overall duration of their career and duration of their career in the current company (in case of self-employed, we asked how long they have been were self-employed), on the amount of weekly worked hours, on the dimension of the company (1–9, 10–49, 50–249 or 250+ employed people), and on their current job classification according to the following definitions: apprentice/consultant, office/manual worker, executive/manager, private practice, other. Finally, patients were asked to provide – with reference to the last 30 days – the number of days they did not work due to migraine, the number of days they worked with reduced ability due to headache, and they were invited to give an estimate of their overall work performance, expressed as percentage on a 1–99% scale (of course referred to the days with reduced ability).

The items tested in this version of the HEADWORK questionnaire, defined on the basis of the results of the previous literature review and patients’ input [23, 25] were revised by a small panel of 12 patients (with both EM and CM) and 8 clinicians that were asked to judge the items and report any problematic issues, namely difficulties in understanding the content of each item. Specifically, patients were asked to judge if in their opinion it was possible to misunderstand what was written, and not if they experienced the phenomenon described in the different items. None of the items was judged as unclear and minor changes were made, in particular: “Solving organizational problems at work” was rephrased from the original “Solving work problems” to focus not on generic outcomes in term of productivity or result of the job done, but on “procedural” issues, i.e. the way in which daily problems with the organization of what has to be done are handled; “Starting a new work task” was rephrased from the original “Starting to work”, as the first version was felt as too much generic and could be taken in a too much broad sense (e.g. starting a work career).

Once items were finalized, they were grouped into two section. The first section included 13 items addressing different work tasks and work-related activities. Patients were asked to report how much of a difficulty they had with each activity using a five-point response scale, ranging between 1 (no difficulty) and 5 (I cannot do it). Examples of these items include talking and interacting with other people, reading and writing abilities. The second section included 12 items addressing the factors that possibly limit patients’ ability with work-related activities and prevented them to perform these activities. Patients were invited to answer on a five-point scale (ranging between 1-no limitations, and 5-complete limitation): examples of these items include negative attitudes of colleagues and environmental triggers, such as noise or smell. For both the section, items had to be answered thinking back to the last 30 days and. The option “not applicable” was left in case an activity or a factor was not of relevance for respondents’ jobs or was not part of the workplace features. There are different reasons for choosing a 5-point scale. First, odd number of items’ response enable to use the central value for those cases in which patients feel a non-mild impact but do not wish to move closer to the maximum value: as we did not use an “agree vs. not agree” scale, we did not carry the risk of leaving a central option meaning “not taking position”. Second, we preferred the 5-item options as it is easier to fill in compared to the 7-item one. In fact, in the 7-item option there are two steps between the lowest (or highest) value and the central one, which are difficult to label. Third, we intended to ground our measure on the definition of disability endorsed by the ICF [24] and operationalized with the WHODAS i.e. limitation in carrying out daily activities due to the presence of a health condition. The content of HEADWORK is close to such a definition, as the activities that are limited by migraine headaches are work-related ones.

The MIDAS [27, 28] is composed of seven questions referred to the preceding three months. The first two questions investigate the impact of headache on work, in terms of missed workdays and of work with at least half reduction, the third and fourth apply the same scheme to household work, and the fifth addresses missed leisure/family/social activities due to headache. Responses are given in terms of days with missed or reduced activities. The sixth question is on the number of days with headache and the seventh is on average pain intensity. MIDAS is scored based on the first five question by simply summing up the days: four severity grades are available, i.e. minimal (0–5), mild (6–10), moderate (11–20), and severe (≥21) disability.

The WHODAS-12 is the short version of the original 36-items WHODAS 2.0. It investigates the same domains as the original version (i.e. understanding and communicating, getting around, self-care, getting along with people, life activities, and participation in society), and accounts for 81% of the variation of the original full questionnaire. Patients are asked to respond to 12 questions referred to daily activities, and they report how much of a difficulty they experienced during the previous 30 days, due to their health condition. Answers have to be rated on a 5-point scale (no problems – complete problems/cannot do the activity), and WHODAS-12 score ranges between 0 and 100, with higher scores reflecting greater disability [29].

The MSQ is a migraine-specific measure of health-related QoL [30]. The questionnaire is composed of 14 items that form three scales, namely role-restriction (RR), role-prevention (RP) and emotional function (EF): each scale has a 0–100 score, with lower scores indicating lower health-related QoL. Items refer to different daily activities or social situations, and patients have to rate how frequently migraine determined an impact on these activities, thinking back to the previous four weeks, using a 6-point scale from never to always. The MSQ has mostly been used with patients with EM, but it has also been validated in those with CM [31, 32].

### Data analyses

Continuous variables were reported as means and standard deviations (SD), categorical variables as frequencies and percentages. Data were analyzed with SPSS 19.0.

#### Factor structure and item reduction

The approach to the definition of the HEADWORK questionnaire’s factor structure involved an exploratory factor analysis (EFA) on 70% of the sample, followed by a confirmatory factor analysis (CFA) on the remaining 30% and, later on, on the whole sample.

Prior to carrying out the EFA, we transformed the “not applicable” items into missing and evaluated symmetry indexes: items that were clearly asymmetric (i.e. with symmetry index ≥2.58) were eliminated from the dataset. We also looked at the inter-correlation between items, separately within the two sections of HEADWORK, and removed those items with an overall inter-item correlation index below .300. We also removed those items that showed correlation indexes >.800 with at least two other items in each of the two sections of HEADWORK (i.e. more than 10% of the total number of items), to avoid, or at least limit, the risk of multicollinearity or singularity problems [33]. Suitability of data for factor analysis was assessed with Bartlett’s test of sphericity (BTS), adequate if *P* < .05 [33], and with Kaiser-Meyer-Olkin Measure of Sampling Adequacy (KMO), adequate if > 0.70 [33, 34]. EFA was carried out using principal component extraction and direct Oblimin rotation to extract data, as we reasonably expected that, should EFA define more than one factor, they might have been significantly correlated each other.

Three steps to item reduction were followed. First, we deleted items that did not load into any factor (i.e. with factor loadings <.400) as they gave no contribution to questionnaire’s structure. Second, items loading into more than one factor (i.e. with factor loadings >.40) were deleted as they would determine high instability to the factor structure of the questionnaire. Third, we looked at scale reliability information, namely inter-item correlation, item-total correlation and Cronbach’s Alpha: we deleted items that were either too much correlated (i.e. coefficient > .800) with at least two other items, or that showed a low item-total correlation (i.e. coefficient < .300), or that would make Alpha increase if deleted.

The ratio between Chi^2^ and degrees of freedom (Chi^2^/d.f.) and the Root Mean Square Error of Approximation (RMSEA) were used as model fit indices for CFA: Chi^2^/d.f. < 3 and RMSEA< 0.08 were considered acceptable [34].

#### Internal consistency

Internal consistency was assessed using Cronbach’s Alpha coefficient, item-total correlation after correcting for overlap (i.e. removing the item from the total score), and the average inter-item correlation. Scales were considered to have a good reliability if Cronbach’s alpha was >.70 [35], if item-total correlation indexes were > 0.40, and if average inter-item correlations were comprised between 0.30 and 0.70 [36].

#### Construct validity

Construct validity was tested in different ways. First, by correlating the two HEADWORK scales with WHODAS-12, MSQ, and MIDAS sores, headache frequency, and average pain severity in the previous three months, the amount of lost workdays and of workdays in which productivity was impaired, and with the estimated average productivity in days worked with reduced productivity. We used Pearson’s correlation, and expected that HEADWORK scales: a) were directly correlated with all the other variables (with the exclusion of MSQ scores, and with the estimated average productivity, where an inverse correlation is expected), and with correlation coefficients < .700; b) had a stronger correlation with the WHODAS-12 than with the MSQ scores, as the construct underlining HEADWORK is the amount and severity of difficulties with work-related activities; c) had a stronger correlation with the average pain severity and the average work ability than with the variables related to frequency of headache, workdays lost and days worked with reduced productivity. Significance was set with alpha = 0.0023 after Bonferroni correction and two-tailed testing.

The second approach to the evaluation of construct validity, was made by testing the differences in HEADWORK scales between males and females, between patients with EM and CM, between patients working more than 40 h/week and those working less, and between patients employed in medium/large companies and those employed in small ones. We used Student’s t-test and expected that females, patients with CM, those working more than 40 h/week and those employed in larger companies might experience higher difficulties. Significance was set with alpha = 0.0125 after Bonferroni correction and two-tailed testing. We also tested HEADWORK scales across patients with different degrees of education, and across patients with different types of contract using One-Way ANOVA and Bonferroni post-hoc test. We expected that those with lower education and those with higher-level positions (i.e. employers, private practitioners or people with executive roles vs. those with temporary jobs and office/manual workers) might experience higher difficulties.

For cross-sectional comparisons, Hedges’ *g* was used as a measure of effect size (ES): ES around or higher than 0.5 indicate a medium effect; ES around of higher than 0.8 indicate a large effect. Data were expressed as means and 95% Confidence Intervals (95% C.I.).

## Results

### Sample description

A total of 377 patients were enrolled in the study. However, four records showed important incompleteness of HEADWORK questionnaire, and further eleven did not have complete MIDAS, WHODAS-12 or MSQ. Therefore, 373 questionnaires were used to address the factor structure of HEADWORK, and 362 to address measurement properties. Table 1 reports the main socio-demographic information of the validation sample (*N* = 373). Most of enrolled patients (280) had EM, and most of them (240) had EM without aura. The remaining 93 patients had CM, and most of them (71) had comorbidity to MOH. None of the EM patients had MOH. On average, it was a highly educated sample, as 41.8% completed university studies and mostly composed of females (81%). On average, in the previous month patients with EM lost one day of work and worked 5–7 days with migraine (approximately with 55–60% of their ability), while those with CM lost 3–4 days and worked 14–17 days with migraine (approximately with 50% of their ability). MIDAS scores, days with headache and WHODAS-12 scores were higher among those with CM than in those with EM, indicating higher a disability level, while MSQ scores were lower, indicating a lower quality of life.

**Table 1:** Sociodemographic characteristics of the HEADWORK validation sample

	EM (*N* = 280)	CM (*N* = 93)	All patients (*N* = 373)
Female gender	224 (80%)	78 (83.9%)	302 (81%)
Age	42.1 ± 10.0	44.0 ± 9.2	42.6 ± 9.8
Education level			
Up to secondary	39 (13.9%)	18 (19.4%)	57 (15.3%)
High	109 (52.4%)	51 (54.8%)	160 (42.9%)
University degree or higher	132 (47.1%)	24 (25.8%)	156 (41.8%)
Company size			
Small (1–49 employed)	108 (38.7%)	35 (37.6%)	143 (38.3%)
Medium-Large (50+ employed)	171 (61.1%)	58 (62.4%)	229 (61.7%)
Type of contract			
Stage/Other temporary	16 (5.7%)	3 (3.2%)	19 (5.7%)
Office/Manual worker	186 (66.4%)	63 (67.7%)	249 (74.3)
Executive/Manager	9 (3.2%)	5 (5.4%)	14 (4.2%)
Private Practice/Employer	37 (13.2%)	16 (17.2%)	53 (15.8%)
Duration of career	19.1 ± 10.5	21.3 ± 10.9	19.7 ± 10.6
Career in the present company	13.2 ± 9.8	14.1 ± 9.8	13.5 ± 9.8
Weekly worked hours	39.3 ± 10.8	36.9 ± 9.9	38.9 ± 10.6
Workdays lost in the previous month	1.0 ± 2.0	3.8 ± 6.0	1.7 ± 3.6
Days worked with headache in the previous month	6.5 ± 5.7	16.8 ± 7.8	8.8 ± 7.6
Average productivity (%)	56.4 ± 23.0	51.6 ± 21.0	55.1 ± 22.7
MIDAS score	27.5 ± 23.8	101.3 ± 60.3	41.8 ± 45.3
Days with headache/3 months	18.9 ± 11.3	61.7 ± 15.6	27.7 ± 21.2
Average pain intensity	7.2 ± 1.7	7.8 ± 1.6	7.3 ± 1.7
WHODAS-12	24.6 ± 17.0	41.2 ± 19.6	28.0 ± 18.7
MSQ-RR	51.6 ± 20.3	30.2 ± 18.9	47.2 ± 21.8
MSQ-RP	65.3 ± 22.0	43.2 ± 22.3	60.8 ± 23.7
MSQ-EF	62.9 ± 27.8	34.4 ± 27.7	57.1 ± 30.0

*Notes. EM* episodic migraine, *CM* chronic migraine, *MOH* medication overuse headache, *MIDAS* migraine disability assessment, *WHODAS-12* 12-items WHO disability assessment, *MSQ* migraine specific quality of life questionnaire, *RR* role restriction, *RP* role prevention, *EF* emotional function

### Factor analysis

Additional file 1: Tables S1-S4 report the results of items’ distribution with regard to the amount of not applicable ones and, after transformation into missing, of inter-item correlation and asymmetry, as well as the full inter-item correlation matrix. Among those of the first section of the preliminary version of HEADWORK, two items were excluded from the EFA: Managing work stress, due to high asymmetry; Reaching the workplace due to high correlation (>.800) with two other items (Moving from one place to another; Driving a car). From the second section, five items were deleted as they showed high asymmetry: Having to work on shifts rotation; Side effect of symptomatic drugs; Side effect of prophylactic drugs; Feeling dazed/numb; Work stress.

The EFA carried out on 70% of the sample showed that, for both the first and the second section of the HEADWORK Questionnaire, a single factor was found: with regard to the first section, it explained 68.1% of the variance of the questionnaire, while for the second section it explained 49.9%. The CFA, carried out on the remaining 30% of the sample confirmed the factor structure for the first section of HEADWORK, with a similar amount of explained variance (64.7%) and adequate fit indices. With regard to the second section, the scale had an average inter-item correlation that was not satisfactory (.379) and one of the item that was previously included (Need to take an excessive amount of symptomatic drugs) was critical. It showed inadequate factor loading (.353) and its elimination made Cronbach’s Alpha to increase. It was therefore deleted, and the new CFA showed better fit indices, including a higher average inter-item correlation (.423) and explained a higher proportion of variance (53% instead of 47.8%). Additional file 1: Tables S5-S9 show the full factor structure and the reliability analysis for both sections, separately for EFA and CFA.

Table 2 reports the results of the factor analysis carried out over the whole sample. Both sections were composed of one factor, which accounted for 67.1%, and 52.1% of variance, respectively, with good internal consistency and fit indices. Thus, the first HEADWORK scale, which we named “Work-related difficulties”, is composed of eleven items with a theoretical range 11–55: actually, it ranged between 11 and 53 and its mean was 31.4 (SD 9.0). The second HEADWORK scale, which we named “Factors contributing to work difficulties”, is composed of six items with a theoretical range 6–30: actually, it ranged between 6 and 29 and its mean was 15.8 (SD 5.0).

**Table 2:** Factor analysis and reliability data of HEADWORK questionnaire (*N* = 373)

	Loadings	Item Mean ± SD	Item-Total Correlation	Alpha if item excluded
Section A: Work-related difficulties KMO= .938; BTS, *P*<.001; Eigenvalue: 7.377 (67.1% of variance) Alpha= .950; Inter-item R= .636; Average Item-total R= .775 Chi2= 131.2; d.f.= 44; Chi2/d.f.= 2.98; RMSEA=0.047				
Paying attention to work tasks	.883	2.94 ± 0.97	.851	.942
Solving organizational problems at work	.876	2.96 ± 1.04	.832	.943
Starting a new work task	.865	2.87 ± 1.03	.818	.943
Dealing with work problems	.851	2.95 ± 0.96	.830	.943
Reading and writing	.822	2.90 ± 1.05	.784	.945
Using the PC	.795	3.13 ± 0.98	.753	.946
Answering the phone	.765	2.67 ± 1.01	.754	.946
Driving a car	.762	2.81 ± 1.13	.745	.946
Moving from one place to another	.760	2.75 ± 1.07	.741	.946
Talking and interacting with other people	.756	2.86 ± 0.88	.743	.946
Understanding what is said	.684	2.46 ± 0.99	.677	.949
Section B: Factors contributing to work difficulties KMO = .830; BTS, *P* < .001; Eigenvalue: 3.127 (52.1% of variance) Alpha = .808; Inter-item *R* = .412; Average Item-total *R* = .570 Chi^2^ = 20.1; d.f. = 9; Chi^2^/d.f. = 2.23; RMSEA = 0.050				
Noise in the workplace	.840	2.94 ± 1.06	.715	.744
Smell in the workplace	.767	2.58 ± 1.20	.627	.762
Brightness of workplace	.765	2.75 ± 1.07	.676	.752
Extended working hours	.652	2.72 ± 1.10	.579	.774
Negative attitudes of colleagues	.428	2.17 ± 1.04	.424	.806
Air conditioning	.419	2.19 ± 1.15	.400	.809

*Notes. KMO* Kaiser-Meyer-Olkin Measure of Sampling Adequacy, *BTS* Bartlett’s Test of Sphericity, *d.f.* degrees of freedom, *RMSEA* Root Mean Square Error of Approximation, *SD* standard deviation

### Construct validity

Table 3 reports the results of correlations between HEADWORK scales and WHODAS-12, MSQ, MIDAS scores, headache frequency and average pain severity in the previous three months, the amount of lost workdays in the previous month, the amount of workdays in which productivity was impaired and the estimated average productivity in those days. All correlations were significant, with the exception of the first HEADWORK scale (Work-related difficulties), and the number of days with reduced productivity, and all correlations were in the expected direction. As expected, HEADWORK scales were more strongly correlated with the WHODAS-12 than with MIDAS and MSQ scores and also with the average pain severity and the average work ability rather than with frequency of headache, workdays lost and days worked with reduced productivity.

**Table 3:** Correlation analysis between HEADWORK scales, patient-reported outcomes, headache frequency, pain intensity and productivity indexes (*N* = 362)

	Work-related difficulties	Factors contributing to work difficulties
WHODAS-12	.607*	.565*
MSQ-RR	−.593*	−.537*
MSQ-RP	−.586*	−.522*
MSQ-EF	−.496*	−.465*
MIDAS	.443*	.394*
Headache Frequency/3 months	.247*	.296*
Average pain severity	.367*	.301*
N. of lost workdays	.256*	.206*
N. of days worked with hampered productivity	.123	.220*
Average productivity	−.522*	−.342*

*Notes.* *P < .0023. *WHODAS-12* 12-items WHO disability assessment, *MSQ* migraine specific quality of life questionnaire, *RR* role restriction, *RP* role prevention, *EF* emotional function, *MIDAS* migraine disability assessment

Table 4 reports the results of Student’s *t-*test in assessing the differences in HEADWORK scales between males and females, between patients with EM and CM, between patients working more than 40 h/week and those working less, and between patients employed in medium/large companies and those employed in small ones. Consistently with our expectations, females and patients with CM showed higher scores at both HEADWORK scales, than males and EM patients, with medium to large ES. Contrary to our expectations, people working less than 40 h per week showed higher scores than those working less only at HEADWORK “Factors contributing to work difficulties” scale, with a small ES, while no differences were found for the subscale “Work-related difficulties”. With regard to company size, the results were in line with our expectation, but no significant differences were detected. Finally, the results of One-Way ANOVA testing HEADWORK scales across patients with different degrees of education, and across patients with different types of contract showed no significant differences, also in this case contrary to our expectation.

**Table 4:** Independent sample t-test assessing differences in HEADWORK scales based on gender, migraine type, amount of weekly worked hours and company size

Variable			Mean (95% CI)	t-test (*P*-value)	ES
Gender	Work-related difficulties	Males (*N* = 71)	27.1 (25.2–29.1)	4.41 (<.001)	0.59
		Females (*N* = 302)	32.3 (31.3–33.4)		
	Factors contributing to work difficulties	Males (*N* = 71)	12.8 (11.7–13.9)	5.66 (<.001)	0.77
		Females (*N* = 302)	16.5 (15.9–17.0)		
Migraine type	Work-related difficulties	EM (*N* = 280)	30.5 (29.5–31.6)	3.43 (.001)	0.45
		CM (*N* = 93)	34.5 (32.5–36.5)		
	Factors contributing to work difficulties	EM (*N* = 280)	15.2 (14.6–15.8)	4.57 (<.001)	0.59
		CM (*N* = 93)	18.1 (17.1–19.0)		
Amount of weekly worked hours	Work-related difficulties	Up to 40 h/week (*N* = 247)	31.7 (30.6–32.9)	1.20 (.23)	0.13
		> 40 h/week (*N* = 115)	30.5 (28.8–32.2)		
	Factors contributing to work difficulties	Up to 40 h/week (*N* = 247)	16.2 (15.6–16.9)	2.61 (.009)	0.28
		> 40 h/week (*N* = 115)	14.8 (13.9–15.7)		
Company size	Work-related difficulties	Up to 49 employees (*N* = 143)	30.1 (28.6–31.6)	2.01 (.045)	0.22
		50+ employees (*N* = 229)	32.1 (30.9–33.3)		
	Factors contributing to work difficulties	Up to 49 employees (*N* = 143)	15.1 (14.3–16.0)	1.78 (.075)	0.20
		50+ employees (*N* = 229)	16.1 (15.4–16.8)		

*Notes. 95% CI* 95% Confidence Interval, *EM* episodic migraine, *CM* chronic migraine. Significance set with alpha = 0.0023 after Bonferroni correction

## Discussion

With this paper we present the validation of the HEADWORK questionnaire, a 17-item PROM specifically designed to assess the impact of EM and CM on work-related tasks and the factors that may contribute to such difficulties. Our results showed that the different dimensions regarding the negative influence of migraine on work activities, i.e. the amount and severity of difficulties in work-related tasks and the factors that impact over them, can be measured by two distinct scales. The first scale, named “Work-related difficulties”, is composed of eleven item dealing with the degree to which migraine headaches determine a difficulty in general skills, such as solving organizational problems or starting a new work task, or in specific tasks, e.g. using the computer or talking and interacting with other people. The second scale, named “Factors contributing to work difficulties” is composed by six item, and addresses the degree to which some factors, such as noise of brightness of workplace, or the attitudes of colleagues, negatively impact on difficulties with work-related tasks. Thus, with the validation process, we reduced the amount of items from the initial number of 25 to the final number of 17, and both HEADWORK subscales showed good measurement properties, with higher scores being associated to higher impact levels.

The assessment of migraine-related impact on work-place activities is a relevant research and healthcare topic because migraine is recognized as one of the most burdensome diseases [37–42]. Of notice, the studies published on the Global Burden of Disease (GBD) confirmed that migraine is more prevalent among females and in both sexes in the most productive age, and acknowledged migraine as the seventh position in the rank of top causes of Years Lived with a Disability (YLDs) in 2010, and then to the sixth in 2013, and eventually to the second in 2016 [37–39]. Such an increased in GBD ranking is likely due to the fact that MOH was kept distinct from migraine in the first GBD reports, while in the newly published GBD study, the burden of MOH was partly assigned to migraine and partly to tension-type headache, with the result that migraine ascended to the second rank in the causes of YLD, being responsible for 5.6% of all YLDs [41]. The reasons for such a change are shareable, as MOH is a complication of a pre-existing headache disorder and it does not occur otherwise [42, 43]. Thus we think that it is correct to assume that there is continuity in terms of disease burden between EM and CM with MOH, the impact over work-related tasks being the main domain for negative impact [44, 45]. Our results are in line with such a hypothesis: HEADWORK scores were higher in CM patients for both scales, as compared to their episodic counterpart (with medium to large ES): these findings can be explained by different aspects characterizing CM patients, i.e. higher headache frequency, more severe pain intensity, but mainly the presence of MOH, which in fact was present in most patients of this group. MOH is present in more than 60% of CM patients, as shown by previous literature findings [9–12] and is presumed to be a concause of CM development [13], but is a distinct feature as not all CM patients present with MOH. Mixing primary and secondary headaches may be problematic but, in our opinion, the problem is mostly taxonomic, as HEADWORK is intended to capture work-related difficulties due to the presence of EM and CM, irrespectively from the presence of MOH. It has also to be noted that the single item of the preliminary version of our questionnaire addressing MOH (i.e. Need to take an excessive amount of symptomatic drugs) was not retained in the final version: so, we believe that HEADWORK can be used by both the two subgroups of CM patients, with and without MOH.

HEADWORK is intended to fill in the existing gap on the measurement and better understanding of reduced productivity, a task that presents relevant challenges. As far as we know, three are the main available instrument for this task: the Migraine Work and Productivity Loss Questionnaire (MWPLQ) [46, 47], the MIDAS, and the Work Productivity and Activity Impairment (WPAI) [48]. The MWPLQ is the only migraine-specific tool to assess difficulties in the workplace [46, 47]. It is aimed at measuring the impact of migraine headache on work, in terms of hours of work lost or of hours worked with migraine symptoms, which also includes a set of questions assessing the different activities and influencing factors. These items included difficulty in getting to work, working in proximity to environmental triggers of migraine symptoms, difficulty in handling physical aspects of jobs, visual tasks, mental aspects, and interpersonal issues at work. A grading of limitations in each investigated activity is required, on a 6-point scale, from “no difficulty” to “so much difficulty couldn’t do at all”. This questionnaire was developed to assess the positive impact of acute medications: all questions are focused on in the most recent headache attack, and some of them specifically ask the number of hours missed before and after the medication was taken. The MIDAS includes two questions on the number of days with total or partial impairment in work activities experienced in the previous three months. The question on days with 50% or more impairment in work activities does not allow to capture the whole range of possible productivity reduction which may be lower than 50% [14, 15, 49]. Addressing the full range of limitation is more relevant than missed workdays in migraine patients, as it is the main driver of migraine cost and burden [14, 15, 20–22, 45]. Furthermore, the value of MIDAS seems problematic in those patients with high frequency migraine and CM, because patients are likely to approximate responses to MIDAS questions by multipliers of 5 or 10 [21]. Finally, the WPAI is a generic instrument addressing the negative impact of different diseases on work productivity [48]. Questions of the WPAI investigate the number of lost working hours and of hours worked with partial productivity limitations (as assessed on a 10 point scale) in the past seven days, and includes two questions inquiring how much did the underlining health condition affected productivity while working – as well as it affected other regular daily activities – on a 10-point scale (from “no effect” to “completely prevented from working”). A migraine-specific version of this tool can be found on the developer’s website [50] and its use has been suggested by recent guidelines for randomized trials in CM [51].

In synthesis, the different available PROMS that can be used in migraine patients are not comparable to HEADWORK, as none of them systematically enable to address a set of activities that are relevant to carrying out work-related tasks. The MWPLQ includes some “qualitative” information on the different types of activities and on influencing factors in the work-place, and both the MWPLQ and the WPAI include questions on the degree of impairment while continuing to perform work activities with migraine. However, the MWPLQ has the specific aim to assess difficulties in relation to the use of an acute medication: therefore, the main focus is the amount of time with difficulties in productivity before and after the intake of medication during a single migraine attack. Despite the WPAI was recently used to address the role of nausea and vomiting in determining the economic burden of migraines [52] and in a RCT on the anti-CGRP antibody fremanezumab in CM (data reported at the 2017 International Headache Congress [53]), it has never been formally validated for migraine patients to date. Finally, the time-frame of reference of these questionnaires may be too long (such as the three-month period for MIDAS which may determine reporting bias, particularly in CM patients [21]) or too short (such as the most recent headache episode for MWPLQ, and the previous seven days for WPAI) in order to assess clinically meaningful data for epidemiological and outcome research. On the contrary, the HEADWORK questionnaire is likely to give an appropriate insight on the different dimensions of work-place difficulties in subjects with migraine in a clinical relevant period of time (one month). It addresses not only the degree of work-related limitations, but also the impact on specific work tasks, and the evaluation of whole range of possible degree of impairment (by a scale from “no difficulty” to “I cannot do it”), thus offering an evaluation of the reduced work productivity while experiencing a migraine episode, which is the most relevant driver of the total costs of migraine [14, 15, 19]. In reason of these features, we recommend it is used as a measure of migraine impact over work activities, to produce work-related disability weights in studies evaluating the burden of EM and CM, and as a secondary outcome measure in clinical research.

Some of the results we found were expectable and represent a confirmation of the content of HEADWORK items. Among these, our study confirmed that women and CM patients showed a higher difficulties in work activities and reported more factors contributing to these difficulties than men and episodic migraine patients. In addition to this, the fact that HEADWORK questionnaire showed higher correlation indexes with the WHODAS-12 than with the MSQ and with the MIDAS, was expected in consideration of the similarity in the formulation of item and questionnaire construct.

Other results shed light on the value and novelty of HEADWORK as a measure of impact on work-related activities under different aspects. First, the fact that headaches frequency showed higher correlation with the scale “Factors contributing to work difficulties” than with the scale “Work-related difficulties”. Second, the fact that headache intensity showed higher correlations than headache frequency with HEADWORK scales. This is somehow novel, as one could expect that the presence of an higher number of headaches is a factor associated to the presence of more difficulties, while pain intensity is generally considered as a secondary outcome. Third, the fact that both HEADWORK scales showed little correlation with the number of lost workdays and with the number of days worked with reduced productivity. This aspect constitutes a step forward in the understanding of migraine impact, because previously used parameters, such as absenteeism and presenteeism, may provide only an indirect information on the extent of work-related difficulties: what cannot be inferred with such indirect procedures is the extent of reported limitations with reference to the specificity of the task constituting one’s own work duty. HEADWORK fills in this gap, and the little correlation with commonly used indicators, such as the number of lost workdays and the number of days worked with reduced productivity, is a proof of the fact that the content of HEADWORK is not transposable with them. The strong correlation between the HEADWORK scales and the self-reported productivity in the days worked with reduced ability is a further confirmation of the unique information produced by HEADWORK questionnaire. In our opinion, all of these aspects show the ability of HEADWORK to disentangle the problems due to migraine as a disease – which is accompanied by an ensemble of socio-cultural representation, such as the need to use drugs to function, and stigma (which is particularly affected by the ability to work [54]) – and the presence of single headaches, which may have a “more or less” severe impact depending on several factors. These factors can be connected to the subjective response to therapies, but also to the features of the context in which the person works, in terms, for example, of environmental triggers (like noise or light) or of possibility to quit working or attitudes of colleagues.

Some limitations have to be acknowledged. Sample size was wide enough, as showed by KMO and BTS, but was entirely derived from specialty headache centers: the primary effect of this was the high presence of patients with CM (around 25%) compared to what could be expectable based on the epidemiological presentation of this condition. Second we did not test the stability of the questionnaire, i.e. whether few days after the first administration patients would report similar responses. Similarly, sensitivity to change, i.e. the degree to which changes in patients’ responses are consistent with changes in the disease profile, was not addressed as a longitudinal design would be needed. Such an aspect is of particular relevance, and might constitute important information for clinicians and patients in the process of decision making on the best therapeutic options. In fact, it will be very interesting to understand what may be the main drivers of HEADWORK scales change, considering the potential role of different variables, such as frequency (which is generally considered as the major outcome measure in headache research – but showed a modest correlation with HEADWORK scores), or severity of headaches, but also presence of treatment-related side effects, particularly such those that may have an important role on work-place activities and productivity, such as somnolence, sedation dizziness or fatigue, and which are relatively common with preventive anti-migraine medications. Third, the questionnaire is not designed to distinguish the impact of migraine on work-related aspects in ictal and interictal phases as patients are required to fill in HEADWORK with reference to the previous 30 days, thus taking into account good and bad days. Fourth, headache diaries were used when available, but we do not have track of how many patients had. Diagnosis was clinical and based on ICHD-3Beta criteria for EM with and without aura and CM with and without MOH: however, we cannot exclude mixed diagnoses, i.e. presence of tension-type headaches, for some cases. Finally, among the next steps to further on implement HEADWORK, the definition of cut-off scores is surely the most relevant one. Further clinical and labor-related aspects would however be needed to perform such a task. Frequency of access to emergency departments, recurrence of relapses into MOH and presence of comorbidities, that have been showed to negatively impact on disability and QoL [55], may be relevant clinical indicators, and presence of disability benefits and – prospectively – risk of unemployment may be relevant labour-related indicators for grading of HEADWORK questionnaire.

## Conclusions

We presented the validation of the HEADWORK questionnaire, a brief questionnaire which addresses the impact of migraine on work-related difficulties in terms of presence, and severity, of difficulties in general and specific skills, and it also addresses the factors contributing to these difficulties, defined as negative impact on work tasks. It has been validated in patients with episodic and chronic migraine and it can be used in all populations of employed persons, either adults or adolescents. HEADWORK is composed of 17 items that are grouped in two scales, both of them with good measurement properties, where higher scores reflect the presence of severe difficulties on one side, and of more interfering factors contributing to these difficulties on the other side.

We propose HEADWORK as a feasible way to produce reliable work-related disability weights in studies evaluating the burden of episodic and chronic migraine in epidemiological and clinical research. Further studies are needed to prove its role as an outcome tool, and its ability to assess the balance between therapeutic effects and side effects of given treatment interventions on work performance and productivity. Future studies are also needed to test the validity of HEADWORK in other headache disorders, such as tension-type headache.

## Additional file

Additional file 1: **Table S1.** Full inter-item correlation on HEADWORK first section (Work-related difficulties). **Table S2.** Items’ average score, percentage of not applicable and missing items, item asymmetry index inter-item correlation for HEADWORK first section (Work-related difficulties). **Table S3.** Full inter-item correlation on HEADWORK section B (Factors contributing to work difficulties). **Table S4.** Items’ average score, percentage of not applicable and missing items, item asymmetry index inter-item correlation for HEADWORK section B (Factors contributing to work difficulties). **Table S5.** EFA on HEADWORK section A (Work-related difficulties). **Table S6.** EFA on HEADWORK section B (Factors contributing to work difficulties). **Table S7.** CFA on HEADWORK section A (Work-related difficulties). **Table S8.** CFA on HEADWORK section B (Factors contributing to work difficulties). **Table S9.** second CFA on HEADWORK section B (Factors contributing to work difficulties). (DOCX 39 kb)

## Abbreviations

95% C.I.: 95% Confidence Intervals
BTS: Bartlett’s Test of Sphericity
CFA: Confirmatory factor analysis
Chi2/d.f.: ratio between Chi2 and degrees of freedom
CM: Chronic Migraine
EF: Emotional function
EFA: Exploratory factor analysis
EM: Episodic Migraine
ES: Effect size
GBD: Global Burden of Disease
ICF: International Classification of Functioning, Disability and Health
ICHD-3Beta: International Classification of Headache Disorders, 3-beta version
KMO: Kaiser-Meyer-Olkin Measure of Sampling Adequacy
MIDAS: Migraine Disability Assessment schedule
MOH: Medication Overuse Headache
MSQ: Migraine Specific Quality of Life Questionnaire
MWPLQ: Migraine Work and Productivity Loss Questionnaire
PROMs: Patient-reported outcome measures
QoL: Quality of life
RMSEA: Root Mean Square Error of Approximation
RP: Role-prevention
RR: Role-restriction
SD: Standard deviation
WHODAS-12: World Health Organization 12-items Disability Assessment Schedule
WPAI: Work Productivity and Activity Impairment
YLDs: Years Lived with a Disability
