# Supplementary material for: Validation of a self-reported instrument to assess work-related difficulties in patients with migraine: the HEADWORK questionnaire
Source: J Headache Pain. 2018 Sep 10;19(1):85. doi: 10.1186/s10194-018-0914-7 (PMC6131677; doi:10.1186/s10194-018-0914-7)
Supplement: Supplementary file 1 — Table S1. Full inter-item correlation on HEADWORK first section (Work-related difficulties). Table S2. Items’ average score, percentage of not applicable and missing items, item asymmetry index inter-item correlation for HEADWORK first section (Work-related difficulties). Table S3. Full inter-item correlation on HEADWORK section B (Factors contributing to work difficulties). Table S4. Items’ average score, percentage of not applicable and missing items, item asymmetry index inter-item correlation for HEADWORK section B (Factors contributing to work difficulties). Table S5. EFA on HEADWORK section A (Work-related difficulties). Table S6. EFA on HEADWORK section B (Factors contributing to work difficulties). Table S7. CFA on HEADWORK section A (Work-related difficulties). Table S8. CFA on HEADWORK section B (Factors contributing to work difficulties). Table S9. second CFA on HEADWORK section B (Factors contributing to work difficulties). (DOCX 39 kb) [file 10194_2018_914_MOESM1_ESM.docx]

**Additional file**

**Title**: Validation of a self-reported instrument to assess work-related difficulties in patients with migraine: the HEADWORK questionnaire.

**Authors**: Raggi A, Covelli V, Guastafierro E, Leonardi M, Scaratti C, Grazzi L, Bartolini M, Viticchi G, Cevoli S, Pierangeli G, Tedeschi G, Russo A, Barbanti P, Aurilia C, Lovati C, Giani L, Frediani F, Di Fiore P, Bono F, Rapisarda L, D’Amico D.

**Journal**: The Journal of Headache and Pain.

**Corresponding Author**: Alberto Raggi, PsyD, PhD. Neurology, Public Health and Disability Unit – Neurological Institute C. Besta IRCCS Foundation. e-mail: [alberto.raggi@istituto-besta.it](mailto:alberto.raggi@istituto-besta.it)

**Table S1: Full inter-item correlation on HEADWORK first section (Work-related difficulties)**

|  | A1 | A2 | A3 | A4 | A5 | A6 | A7 | A8 | A9 | A10 | A11 | A12 | A13 |
| --- | --- | --- | --- | --- | --- | --- | --- | --- | --- | --- | --- | --- | --- |
| A1 | – |  |  |  |  |  |  |  |  |  |  |  |  |
| A2 | .735 | – |  |  |  |  |  |  |  |  |  |  |  |
| A3 | .584 | .600 | – |  |  |  |  |  |  |  |  |  |  |
| A4 | .679 | .658 | .603 | – |  |  |  |  |  |  |  |  |  |
| A5 | .614 | .568 | .558 | .731 | – |  |  |  |  |  |  |  |  |
| A6 | .553 | .589 | .536 | .676 | .582 | – |  |  |  |  |  |  |  |
| A7 | .517 | .544 | .489 | .625 | .570 | **.804** | – |  |  |  |  |  |  |
| A8 | .657 | .640 | .604 | .773 | .676 | .690 | .673 | – |  |  |  |  |  |
| A9 | .636 | .617 | .536 | .732 | .630 | .669 | .631 | .793 | – |  |  |  |  |
| A10 | .612 | .620 | .561 | .752 | .672 | .682 | .666 | .795 | **.830** | – |  |  |  |
| A11 | .552 | .588 | .503 | .619 | .533 | .594 | .571 | .609 | .595 | .623 | – |  |  |
| A12 | .598 | .609 | .527 | .614 | .518 | .610 | .565 | .603 | .560 | .586 | **.829** | – |  |
| A13 | .568 | .575 | .560 | .611 | .540 | .585 | .604 | .624 | .585 | .585 | .763 | **.818** | – |
| Average | .609 | .612 | .555 | .673 | .599 | .631 | .605 | .678 | .652 | .666 | .615 | .620 | .618 |

**Table S2: Items’ average score, percentage of not applicable and missing items, item asymmetry index inter-item correlation for HEADWORK first section (Work-related difficulties)**

| Item | % Not Applicable | Mean (95%CI) | % Missing | Skewness (z-score) | Average inter-item Correlation | Correlation >.800 |
| --- | --- | --- | --- | --- | --- | --- |
| A1) Talking and interacting with other people | 0.3% | 2.87  (2.78-2.96) | 0.5% | -2.21 | .609 | – |
| A2) Answering the phone | 2.4% | 2.68  (2.58-2.78) | 2.9% | 0.02 | .612 | – |
| A3) Understanding what is said | - | 2.48  (2.35-2.58) | 0.3% | -0.16 | .555 | – |
| A4) Dealing with work problems | 0.3% | 2.96  (2.86-3.06) | 0.5% | -2.47 | .673 | – |
| *A5) Managing work stress | 0.5% | 3.27  (3.18-3.37) | 0.5% | **-5.85** | .599 | – |
| A6) Reading and writing | 0.3% | 2.89  (2.78-2.99) | 0.5% | -1.06 | .631 | A7 |
| A7) Using the PC | 4.0% | 3.16  (3.06-3.26) | 5.1% | -2.19 | .605 | A6 |
| A8) Paying attention to work tasks | - | 2.94  (2.84-3.04) | 0.8% | -1.60 | .679 | – |
| A9) Starting a new work task | 0.3% | 2.90  (2.79-3.00) | 1.1% | -.156 | .652 | A10 |
| A10) Solving organizational problems at work | 2.1% | 2.93  (2.82-3.04) | 3.2% | -1.30 | .666 | A9 |
| A11) Moving from one place to another | 2.9% | 2.78  (2.67-2.89) | 4.6% | -0.49 | .615 | A12 |
| *A12) Reaching the workplace | 0.3% | 2.64  (2.52-2.75) | 0.3% | 0.11 | .620 | **A11, A13** |
| A13) Driving a car | 7.4% | 2.85  (2.72-2.97) | 7.8% | -0.17 | .618 | A12 |

Note: % of missing was calculated after the transformation of “not applicable” answers into missing values.

* Items to be deleted before EFA

**Table S3: Full inter-item correlation on HEADWORK section B (Factors contributing to work difficulties)**

|  | B1 | B2 | B3 | B4 | B5 | B6 | B7 | B8 | B9 | B10 | B11 | B12 |
| --- | --- | --- | --- | --- | --- | --- | --- | --- | --- | --- | --- | --- |
| B1 | – |  |  |  |  |  |  |  |  |  |  |  |
| B2 | .324 | – |  |  |  |  |  |  |  |  |  |  |
| B3 | .377 | .275 | – |  |  |  |  |  |  |  |  |  |
| B4 | .438 | .343 | .314 | – |  |  |  |  |  |  |  |  |
| B5 | .319 | .274 | .638 | .332 | – |  |  |  |  |  |  |  |
| B6 | .314 | .264 | .534 | .382 | .718 | – |  |  |  |  |  |  |
| B7 | .414 | .299 | .393 | .347 | .322 | .280 | – |  |  |  |  |  |
| B8 | .359 | .189 | .304 | .394 | .457 | .403 | .607 | – |  |  |  |  |
| B9 | .297 | .418 | .533 | .456 | .407 | .371 | .555 | .448 | – |  |  |  |
| B10 | .398 | .256 | .384 | .406 | .325 | .362 | .618 | .644 | .504 | – |  |  |
| B11 | .455 | .278 | .471 | .344 | .366 | .351 | .577 | .453 | .531 | .673 | – |  |
| B12 | .398 | .489 | .345 | .382 | .314 | .276 | .376 | .252 | .503 | .380 | .541 | – |
| Average | .372 | .310 | .415 | .376 | .406 | .387 | .435 | .410 | .457 | .450 | .458 | .387 |

**Table S4: Items’ average score, percentage of not applicable and missing items, item asymmetry index inter-item correlation for HEADWORK section B (Factors contributing to work difficulties)**

| Item | % Not Applicable | Mean (95%CI) | % Missing | Skewness (z-score) | Average inter-item Correlation | Correlation >.800 |
| --- | --- | --- | --- | --- | --- | --- |
| B1) Air conditioning | 30.0% | 2.22  (2.07-2.36) | 30.3% | 2.47 | .372 | – |
| B2) Negative attitudes of colleagues | 7.7% | 2.25  (2.14-2.36) | 8.0% | 2.44 | .310 | – |
| B3) Need to take an excessive amount of symptomatic drugs | 7.7% | 2.61  (2.49-2.73) | 8.8% | -0.78 | .415 | – |
| *B4) Having to work on shifts rotation | 44.6% | 2.01  (1.84-2.17) | 46.9% | **4.44** | .376 | – |
| *B5) Side effect of symptomatic drugs | 10.1% | 2.20  (2.08-2.32) | 11.5% | **2.92** | .406 | – |
| *B6) Side effect of prophylactic drugs | 23.1% | 1.72  (1.60-1.83) | 27.1% | **8.01** | .387 | – |
| B7) Brightness of workplace | 0.5% | 2.81  (2.70-2.92) | 0.5% | -1.87 | .435 | – |
| B8) Smell in the workplace | 1.9% | 2.57  (2.45-2.69) | 1.9% | -0.27 | .410 | – |
| B9) Extended working hours | 11.9% | 2.77  (2.65-2.89) | 13.1% | -1.87 | .457 | – |
| B10) Noise in the workplace | 1.9% | 2.98  (2.88-3.09) | 2.7% | -2.45 | .450 | – |
| *B11) Feeling dazed/numb | 2.9% | 3.07  (2.97-3.17) | 3.2% | **-4.16** | .458 | – |
| *B12) Work stress | 0.5% | 3.31  (3.04-3.23) | 0.8% | **-4.57** | .387 | – |

Note: % of missing was calculated after the transformation of “not applicable” answers into missing values.

* Items to be deleted before EFA

**Table S5: EFA on HEADWORK section A (Work-related difficulties)**

| Eigenvalue:  % variance: | 7.489  68.1% |  |  |  |
| --- | --- | --- | --- | --- |
| **Factor 1. Alpha= .953; Inter-item R= .646; Average Item-total R= .783** |  | Item  Mean±SD | Item-Total Correlation | Alpha if item excluded |
| A10) Solving organizational problems at work | **.895** | 2.88±1.04 | .863 | .945 |
| A8) Paying attention to work tasks | **.890** | 2.90±0.97 | .858 | .945 |
| A4) Dealing with work problems | **.879** | 2.90±0.97 | .846 | .946 |
| A9) Starting a new work task | **.859** | 2.82±1.03 | .819 | .946 |
| A6) Reading and writing | **.840** | 2.87±1.02 | .801 | .947 |
| A2) Answering the phone | **.804** | 2.60±1.01 | .761 | .949 |
| A13) Driving a car | **.801** | 2.71±1.08 | .760 | .949 |
| A1) Talking and interacting with other people | **.799** | 2.85±0.89 | .755 | .949 |
| A7) Using the PC | **.792** | 3.02±0.96 | .747 | .949 |
| A11) Moving from one place to another | **.776** | 2.66±1.03 | .732 | .850 |
| A3) Understanding what is said | **.723** | 2.43±0.99 | .672 | .952 |

Kaiser-Meyer-Olkin Measure of Sampling Adequacy = .937; Bartlett’s Test of Sphericity, P<.001

**Table S6: EFA on HEADWORK section B (Factors contributing to work difficulties)**

| Eigenvalue  % variance | 3.496  49.9% |  |  |  |
| --- | --- | --- | --- | --- |
| **Factor 1. Alpha= .826; Inter-item R= .404; Average Item-total R= .570** |  | Item  Mean±SD | Item-Total Correlation | Alpha if item excluded |
| B10) Noise in the workplace | **.827** | 2.94±1.00 | .703 | .778 |
| B9) Extended working hours | **.794** | 2.61±1.09 | .663 | .783 |
| B8) Smell in the workplace | **.786** | 2.53±1.17 | .645 | .785 |
| B7) Brightness of workplace | **.765** | 2.67±1.04 | .638 | .788 |
| B3) Need to take an excessive amount of symptomatic drugs | **.653** | 2.49±1.06 | .520 | .807 |
| B2) Negative attitudes of colleagues | **.543** | 2.20±1.06 | .429 | .821 |
| B1) Air conditioning | **.507** | 2.14±1.17 | .396 | .829 |

Kaiser-Meyer-Olkin Measure of Sampling Adequacy = .841; Bartlett’s Test of Sphericity, P<.001

**Table S7: CFA on HEADWORK section A (Work-related difficulties)**

| Eigenvalue  % variance | 7.112  64.7% |  |  |  |
| --- | --- | --- | --- | --- |
| **Factor 1. Alpha= .945; Inter-item R= .610; Average Item-total R= .756** |  | Item  Mean±SD | Item-Total Correlation | Alpha if item excluded |
| A10) Solving organizational problems at work | **.813** | 3.13±1.03 | .760 | .938 |
| A8) Paying attention to work tasks | **.880** | 3.04±0.95 | .834 | .935 |
| A4) Dealing with work problems | **.819** | 3.06±0.92 | .792 | .937 |
| A9) Starting a new work task | **.866** | 2.98±1.04 | .812 | .936 |
| A6) Reading and writing | **.785** | 2.98±1.12 | .755 | .938 |
| A2) Answering the phone | **.743** | 2.83±0.99 | .732 | .939 |
| A13) Driving a car | **.746** | 3.03±1.21 | .711 | .941 |
| A1) Talking and interacting with other people | **.743** | 2.89±0.87 | .726 | .939 |
| A7) Using the PC | **.790** | 3.35±0.97 | .758 | .938 |
| A11) Moving from one place to another | **.780** | 2.94±1.14 | .753 | .938 |
| A3) Understanding what is said | **.697** | 2.52±0.99 | .689 | .940 |

Kaiser-Meyer-Olkin Measure of Sampling Adequacy = .919; Bartlett’s Test of Sphericity, P<.001

Chi^2^= 78.7; df=46.

Fit indices: Chi^2^/df= 1.71; RMSEA= 0.079

**Table S8: CFA on HEADWORK section B (Factors contributing to work difficulties)**

| Eigenvalue  % variance | 3.343  47.8% |  |  |  |
| --- | --- | --- | --- | --- |
| **Factor 1. Alpha= .811; Inter-item R= .379; Average Item-total R= .549** |  | Item  Mean±SD | Item-Total Correlation | Alpha if item excluded |
| B10) Noise in the workplace | **.833** | 2.83±1.21 | .696 | .758 |
| B9) Extended working hours | **.542** | 2.86±1.12 | .566 | .783 |
| B8) Smell in the workplace | **.694** | 2.62±1.26 | .506 | .794 |
| B7) Brightness of workplace | **.891** | 2.87±1.16 | .708 | .757 |
| B3) Need to take an excessive amount of symptomatic drugs | **.336** | 2.52±1.18 | .377 | .815 |
| B2) Negative attitudes of colleagues | **.448** | 2.08±1.07 | .442 | .803 |
| B1) Air conditioning | **.578** | 2.32±1.15 | .550 | .785 |

Kaiser-Meyer-Olkin Measure of Sampling Adequacy = .762; Bartlett’s Test of Sphericity, P<.001

Chi^2^= 23.7; df=14.

Fit indices: Chi^2^/df= 1.69; RMSEA= 0.078

Item B3 should be deleted at CFA since it does not reach the minimum level of factor loading (which is equal to .400) and since its elimination would make the scale’s Cronbach’s Alfa slightly higher. Also, it has to be noted that the average inter-item correlation of the whole scale is below the desired value of .400.

**Table S9: second CFA on HEADWORK section B (Factors contributing to work difficulties)**

| Eigenvalue  % variance | 3.181  53.0% |  |  |  |
| --- | --- | --- | --- | --- |
| **Factor 1. Alpha= .815; Inter-item R= .423; Average Item-total R= .582** |  | Item  Mean±SD | Item-Total Correlation | Alpha if item excluded |
| B10) Noise in the workplace | **.855** | 2.83±1.21 | .751 | .747 |
| B9) Extended working hours | **.514** | 2.86±1.12 | .494 | .805 |
| B8) Smell in the workplace | **.714** | 2.62±1.26 | .575 | .790 |
| B7) Brightness of workplace | **.904** | 2.87±1.16 | .776 | .743 |
| B2) Negative attitudes of colleagues | **.458** | 2.08±1.07 | .450 | .813 |
| B1) Air conditioning | **.491** | 2.32±1.15 | .448 | .814 |

Kaiser-Meyer-Olkin Measure of Sampling Adequacy = .794; Bartlett’s Test of Sphericity, P<.001

Chi^2^= 12.1; df=9.

Fit indices: Chi^2^/df= 1.34; RMSEA= 0.048
